# Association between *IL10* rs1800896 polymorphism and risk of pediatric asthma: A meta‐analysis

**DOI:** 10.1111/crj.13714

**Published:** 2023-11-08

**Authors:** Zhihong Zhou, Hui Zhang, Yuanhong Yuan

**Affiliations:** ^1^ School of Nursing Hebi Polytechnic Hebi China; ^2^ SeHan University Yeongam‐gun Jeollanam‐do Republic of Korea; ^3^ Department of Hepatopathy Hunan Children's Hospital Changsha China; ^4^ Emergency Center Hunan Children's Hospital Changsha China

**Keywords:** asthma, children, IL10, pediatric, polymorphism

## Abstract

**Introduction:**

Asthma is a common chronic condition in children. Several studies have explored the potential association between *IL10* rs1800896 polymorphism and the risk of asthma in children, but the findings have been inconsistent. To address these discrepancies, we conducted a systematic review and meta‐analysis to assess the relationship between *IL10* rs1800896 polymorphisms and the susceptibility to pediatric asthma.

**Methods:**

A literature search was conducted in PubMed, Scopus, Web of Science, and CNKI databases to identify eligible studies through April 2022. Meta‐analysis was then performed using five genetic models: dominant, recessive, homozygous, heterozygous, and allele.

**Results:**

A total of 12 studies comprising 1645 cases along with 1447 controls were included in this meta‐analysis. It was found that rs1800896 was not associated significantly with susceptibility to childhood asthma in all genetic models investigated. Subgroup analysis based on the ethnic background of the subjects revealed that rs1800896 was significantly linked to a lower risk of pediatric asthma among Asians in the homozygous model (OR = 0.311, 95% CI = 0.152–0.637, *P* = 0.001) and in the recessive model (OR = 0.585, 95% CI = 0.405–0.846, *P* = 0.004), whereas no significant relationship was observed in Egyptians (*P* > 0.05).

**Conclusion:**

In conclusion, *IL10* rs1800896 polymorphism may be useful as a predictive marker for childhood asthma in Asians, although further studies are needed to validate the study results.

## Graphical abstract

The association between *IL10* rs1800896 polymorphisms and pediatric asthma risk was evaluated using a meta‐analysis. A total of 12 studies with 1645 cases and 1447 controls were included. It was found that *IL10* rs1800896 polymorphism was not significantly associated with risk of childhood asthma in all genetic models studied, although subgroup analysis revealed that the polymorphism was significantly associated with a lower risk of pediatric asthma in Asians.

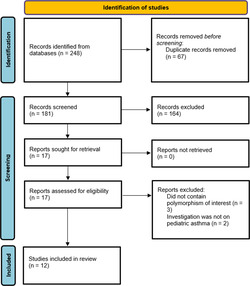

## 1 INTRODUCTION

Asthma is a respiratory disorder that causes respiratory difficulties, a feeling of constriction in the chest, and coughing. It is one of the most common chronic diseases worldwide, affecting about 10% of adults and a larger proportion of children.^1^ The condition is characterized by persistent inflammatory response in the respiratory tract. Since inflammatory processes are primarily coordinated by cytokines, cytokines have been found to be involved in the development of asthma.^2^

One cytokine commonly associated with asthma is interleukin‐10 (IL‐10). IL‐10 is a strong immunomodulatory molecule that suppresses the production of proinflammatory cytokines and promotes B‐cell generation and maturation.^3^, ^4^ Low expression of IL‐10 can contribute to the development of asthma, whereas high levels of IL‐10 can protect against airway inflammation.^5^, ^6^ Therefore, the production of IL‐10 should be controlled to an optimal level.

It is known that cytokine levels can be affected by polymorphisms in their genes, which in turn can affect the risk for a number of disorders.^6^, ^7^, ^8^, ^9^, ^10^, ^11^ Several *IL10* polymorphisms have been reported; in particular, the *IL10* rs1800896 polymorphism has attracted much attention due to its position within a presumed negative regulatory region, known as the binding site for ETS transcription factors. Multiple investigations have been undertaken in different populations to investigate the association between *IL10* rs1800896 polymorphism and asthma in children.^12^, ^13^, ^14^, ^15^, ^16^, ^17^, ^18^, ^19^, ^20^, ^21^, ^22^, ^23^ The majority of the studies employed limited sample sizes, which compromised their statistical power to derive conclusive findings, leading to conflicting results. Therefore, in this study, we conducted a systematic review and meta‐analysis to combine all available published data to investigate whether *IL10* rs1800896 polymorphism contributes to susceptibility to asthma.

## 2 METHODS

### 2.1 Literature search

The electronic databases PubMed, Scopus, Web of Science, and CNKI were searched using the following terms: “(IL10 or interleukin‐10) and (polymorphism) and (asthma).” The search was performed in April 2022, with no language restriction. Search results were deduplicated using EndNote and then screened for eligibility based on title and abstract. Eligibility criteria included: (1) investigated the relationship between *IL10* rs1800896 polymorphisms and risk of pediatric asthma; (2) case–control study; (3) study on human participants, not cell line or animal studies; and (4) genotype or allele frequency data were available in the original text or were extrapolable. Exclusion criteria: (1) non‐original research, such as case reports, meeting abstracts, letters, etc. and (2) did not contain sufficient data.

### 2.2 Data extraction

All data were extracted independently by the two authors using the established search strategies and inclusion criteria. Disagreements were resolved by a third author. Data extracted from the literature included first author, year of publication, country, ethnicity, sample size, and genotype and allele distribution in the case and control groups.

### 2.3 Quality assessment

The quality of the case–control studies was assessed using the Newcastle–Ottawa scale, which evaluates various criteria including participant selection, group comparisons, and measurement of exposure factors. Scores on the Newcastle‐Ottawa scale ranged from 0 to 9, with studies scoring 5 or higher being classified as high‐quality studies.

### 2.4 Statistical analysis

Data from eligible studies were pooled to measure the association between *IL10* rs1800896 polymorphism and pediatric asthma risk using STATA software by estimating odds ratios (ORs) and 95% confidence intervals. Genetic association was measured under different genetic models: homozygous, heterozygous, dominant, recessive, and allele. Heterogeneity between studies was assessed using I2 and P. If there was low heterogeneity, the fixed‐effect model was used. On the contrary, when the heterogeneity was high, a random‐effects model was used. Subgroup analysis by ethnicity was performed to determine whether ethnic background had an effect on genetic susceptibility. To evaluate the presence of publication bias, Egger's test was performed, and a visual examination of the funnel plot was conducted. In addition, we conducted a sensitivity analysis to determine whether the findings were affected by omitting individual studies.

## 3 RESULTS

### 3.1 Study selection

**FIGURE 1:**
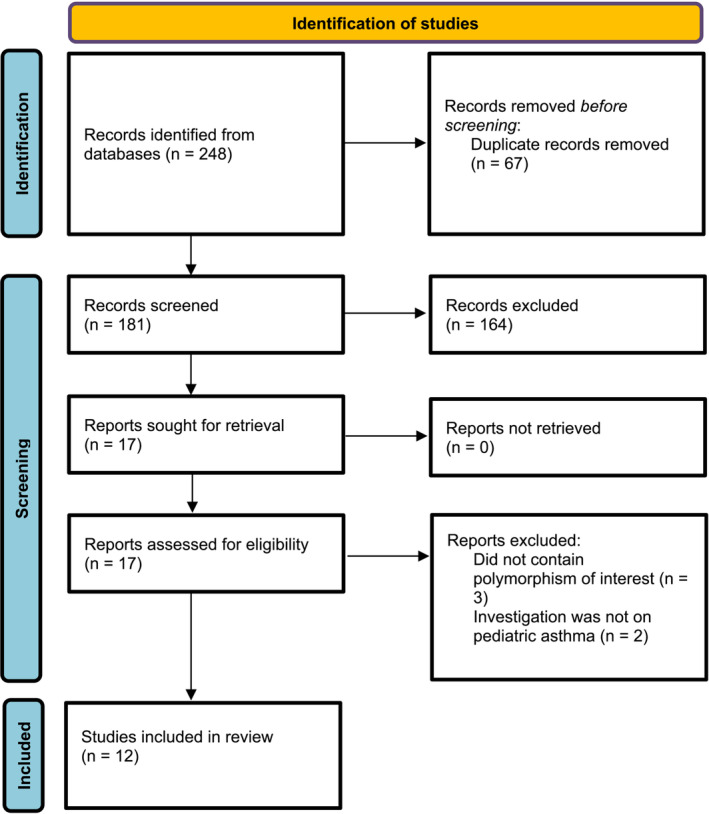
PRISMA flow chart of study selection.

We identified a total of 248 articles from PubMed, Scopus, Web of Science, and CNKI databases. After duplicate entries were removed using EndNote, 181 articles remained to be screened. The initial screening excluded 164 studies that failed to meet the inclusion criteria. Of the remaining 17 articles, the full texts were retrieved. Of these, five articles were further excluded, and 12 studies were incorporated in this study.^12^, ^13^, ^14^, ^15^, ^16^, ^17^, ^18^, ^19^, ^20^, ^21^, ^22^, ^23^ The study selection process is visually presented in Figure 1, which displays the PRISMA flow diagram.

The included reports comprised 1645 cases and 1447 controls (Table 1). Eight studies were conducted in Asians (Chinese, Koreans, Saudi Arabians, and Iranians), while the remaining four studies were conducted in Egyptians. All studied were of high quality according to the Newcastle–Ottawa scale (Supporting Information). Most of the studies did not report the prevalence of patients with different levels of asthma severity and whether the patients had a family history of asthma. For asthma severity, the study by Movahedi et al^12^ comprised 49 mild persistent and 11 moderate persistent asthma patients; the study by Zedan et al^13^ comprised 36 intermittent and 33 persistent asthma patients; the study by Hussein et al^16^ comprised 65 mild, 70 moderate, and 65 severe patients; whereas the study by Maghraby et al^17^ comprised 34 mild, 48 moderate, and 18 severe patients. It is important to note that only Movahedi et al^12^ and Zedan et al^13^ reported genetic association data separately for patients with different levels of asthma severity. On the other hand, data on family history was only provided by Movahedi et al,^12^ Zedan et al,^13^ and Shahin et al.^18^ The three studies respectively reported that 23, 31, and 16 of the patients had a family history of asthma. Despite this, Shahin et al^18^ did not report data on genetic association separately for patients with and without a family history. Given the small number of studies that reported genetic association data separately for different levels of asthma severity and presence of family history, subgroup analysis was not performed for these parameters.

**TABLE 1:** Study characteristics.

Study ID	Country	Method	Ethnicity	Cases					Cases HWE	Controls					Controls HWE
				GG	GA	AA	G	A		GG	GA	AA	G	A	
Li 2007^19^	China	PCR‐SSP	Asian	20	8	2	48	12	0.36	16	7	3	39	13	0.15
Movahedi 2008^12^	Iran	PCR‐SSP	Asian	0	59	0	59	59	<0.01	53	75	12	181	99	0.04
Zedan 2008^13^	Egypt	PCR‐SSP	Others	11	39	19	61	77	0.23	8	85	5	101	95	<0.01
Hussein 2011^14^	Egypt	PCR‐SSP	Others	82	90	48	254	186	0.02	23	54	33	100	120	0.92
Kim 2011^15^	Korea	PCR‐RFLP	Asian	291	40	2	622	44	0.63	215	30	3	460	36	0.02
Tan 2013^20^	China	Sequenom	Asian	134	23	0	291	23	0.32	182	16	0	380	16	0.55
Hussein 2014^16^	Saudi Arabia	PCR‐SSP	Asian	75	86	39	236	164	0.12	8	25	17	41	59	0.81
Xu 2014^21^	China	Sequenom	Asian	161	39	0	361	39	0.13	73	108	8	254	124	<0.01
Shahin 2017^18^	Egypt	qPCR	Others	4	9	17	17	43	0.15	18	2	0	38	2	0.81
Zhang 2018^22^	China	Sequenom	Asian	3	51	151	57	353	0.57	1	39	170	41	379	0.43
Zhen 2018^23^	China	Sequenom	Asian	NA	NA	NA	5	79	NA	NA	NA	NA	3	113	NA
Maghraby 2021^17^	Egypt	PCR‐RFLP	Others	14	20	66	48	152	<0.01	90	4	6	184	16	<0.01

### 3.2 Meta‐analysis findings: Homozygous model

**FIGURE 2:**
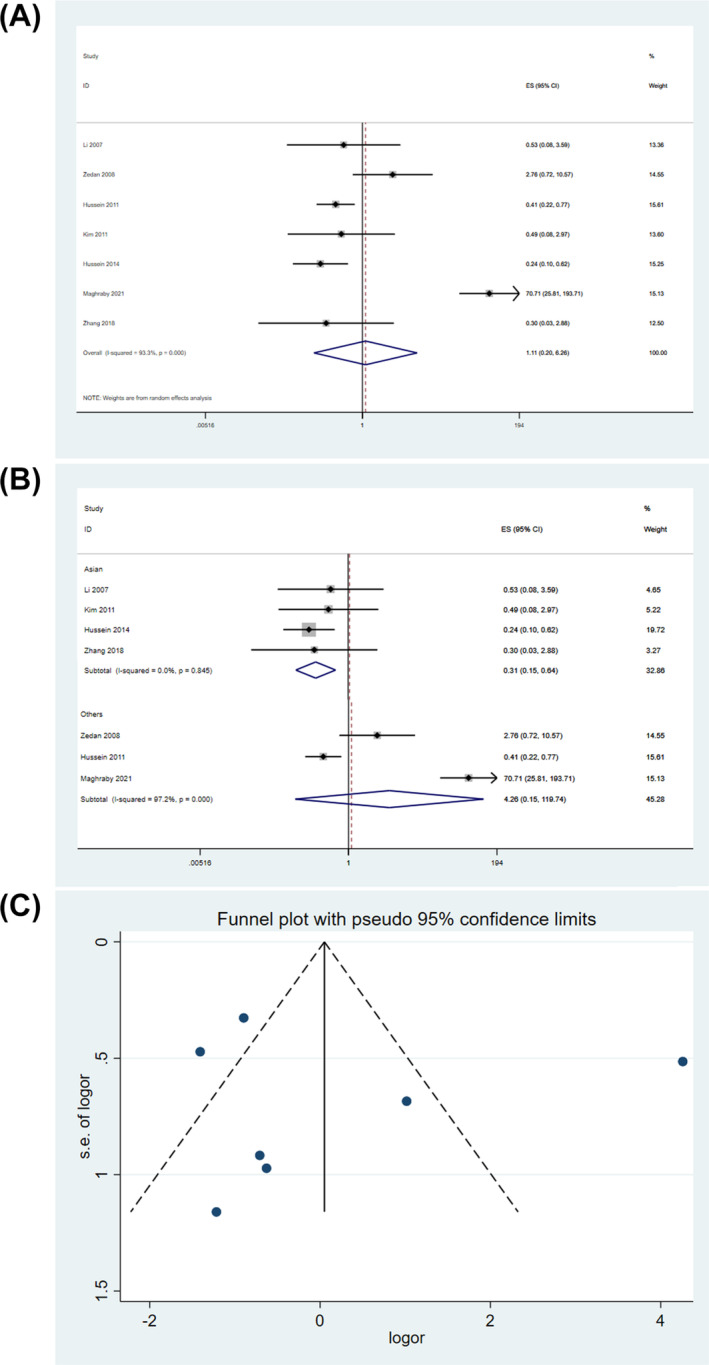
Genetic association in homozygous model. (A) Overall association. (B) Subgroup analysis by ethnicity. (C) Funnel plot.

Pooled data from the studies using a random‐effects model found a lack of significant association between rs1800896 and risk of pediatric asthma in homozygous model (OR = 1.111, 95% CI = 0.197–6.263, *P* = 0.905) (Figure 2A). Sensitivity analysis showed that omitting Maghraby et al^17^ or Zedan et al^13^ reduced the OR value, but the results were still not significant (Supporting Information). Interestingly, subgroup analysis according to the ethnicity revealed that the *IL10* rs1800896 polymorphism was significantly associated with reduced pediatric asthma risk in Asians (fixed‐effect model, OR = 0.311, 95% CI = 0.152–0.637, *P* = 0.001) but no significant association in Egyptians (random‐effects model, *P* = 0.395) (Figure 2B). When evaluated with Egger's test (*P* = 0.833) and the funnel plot inspection (Figure 2C), no publication bias was detected.

### 3.3 Meta‐analysis findings: Heterozygous model

**FIGURE 3:**
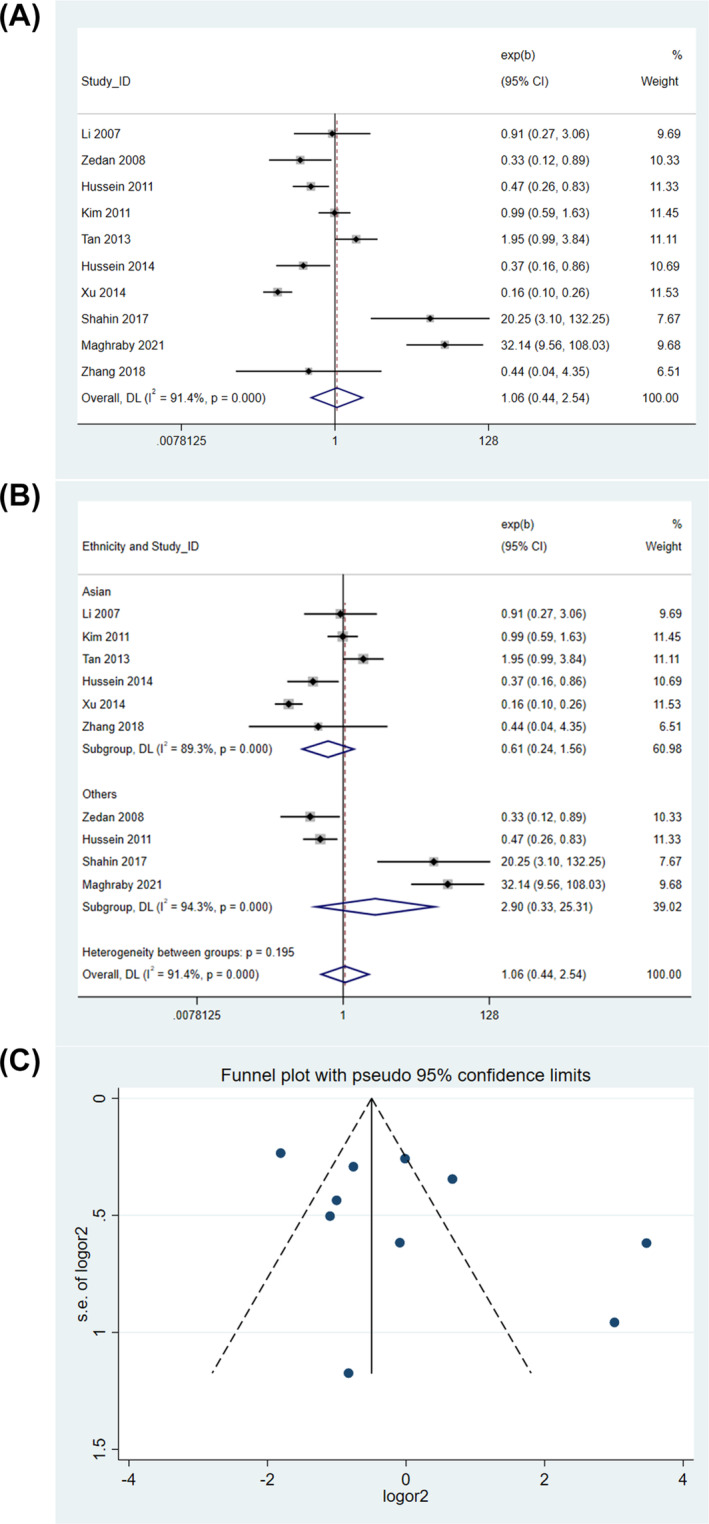
Genetic association in heterozygous model. (A) Overall association. (B) Subgroup analysis by ethnicity. (C) Funnel plot.

In the heterozygous model, no significant association was observed between *IL10* rs1800896 polymorphism and pediatric asthma risk (random‐effects model, OR = 1.063, 95% CI = 0.445–2.541, *P* = 0.890) (Figure 3A). Sensitivity analysis showed that omission of Tan et al,^20^ Shahin et al,^18^ or Maghraby et al^17^ reduced OR to less than 1, but the results were still not significant (Supporting Information). Subgroup analysis by ethnicity revealed no difference between Asians and Egyptians—both groups showed no significant association (Figure 3B). The funnel plot was visually inspected, revealing no signs of asymmetry (Figure 3C), suggesting the absence of publication bias. This observation was further supported by Egger's test (*P* = 0.145), which also indicated no **s**ignificant publication bias.

### 3.4 Meta‐analysis findings: Dominant model

**FIGURE 4:**
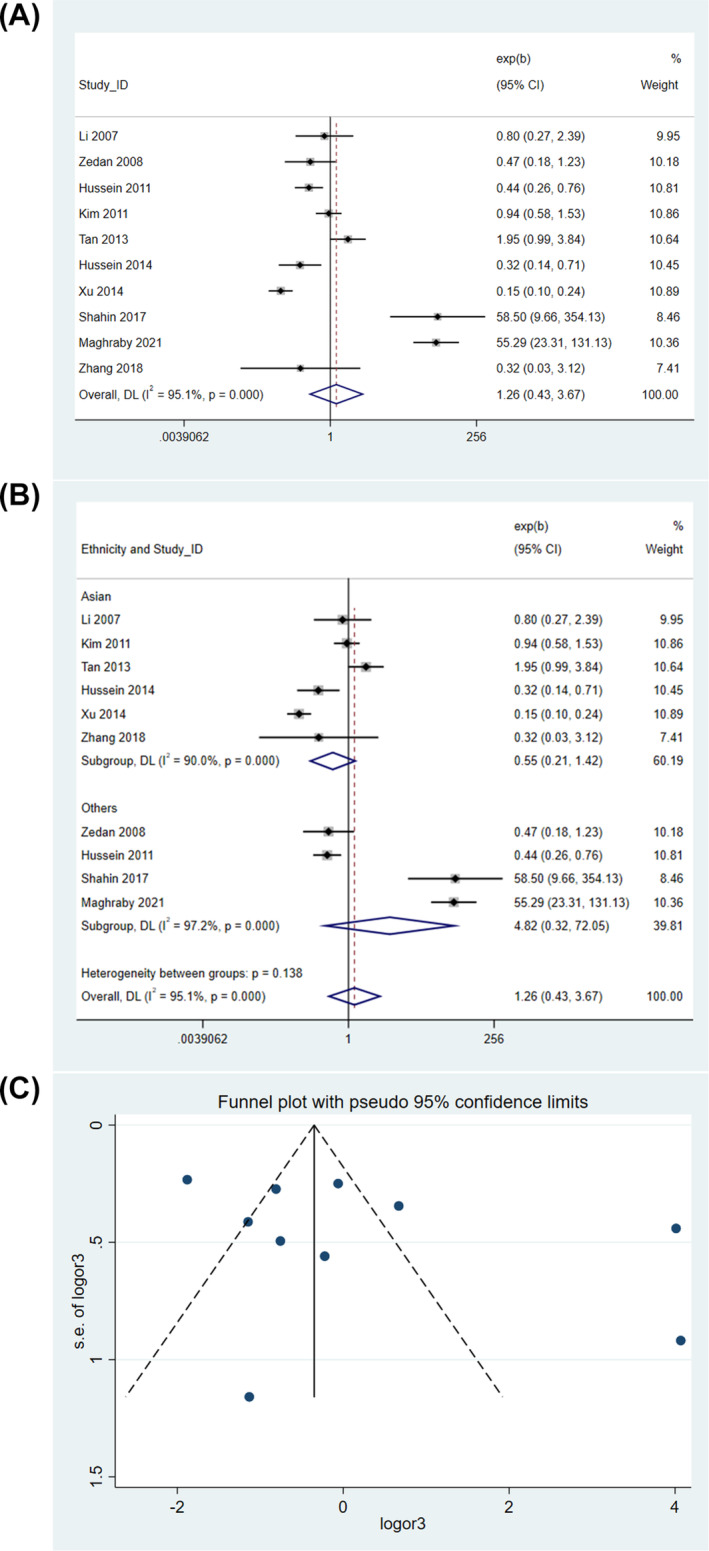
Genetic association in dominant model. (A) Overall association. (B) Subgroup analysis by ethnicity. (C) Funnel plot.

A significant association between *IL10* rs1800896 polymorphism and risk of pediatric asthma was also not observed when the data were analyzed according to the dominant model (random‐effects model, OR = 1.257, 95% CI = 0.430–3.671, *P* = 0.676) (Figure 4A). Sensitivity analysis showed that when data from Shahin et al^18^ or Maghraby et al^17^ were removed from the analysis, the OR became smaller than 1 overall, but the result was still not statistically significant (Supporting Information). Subgroup analysis showed that the two groups, Asians and Egyptians, had no significant association (Figure 4B). Egger's test revealed no publication bias (P = 0.217), consistent with the absence of asymmetry in the funnel plot (Figure 4C).

### 3.5 Meta‐analysis findings: Recessive model

**FIGURE 5:**
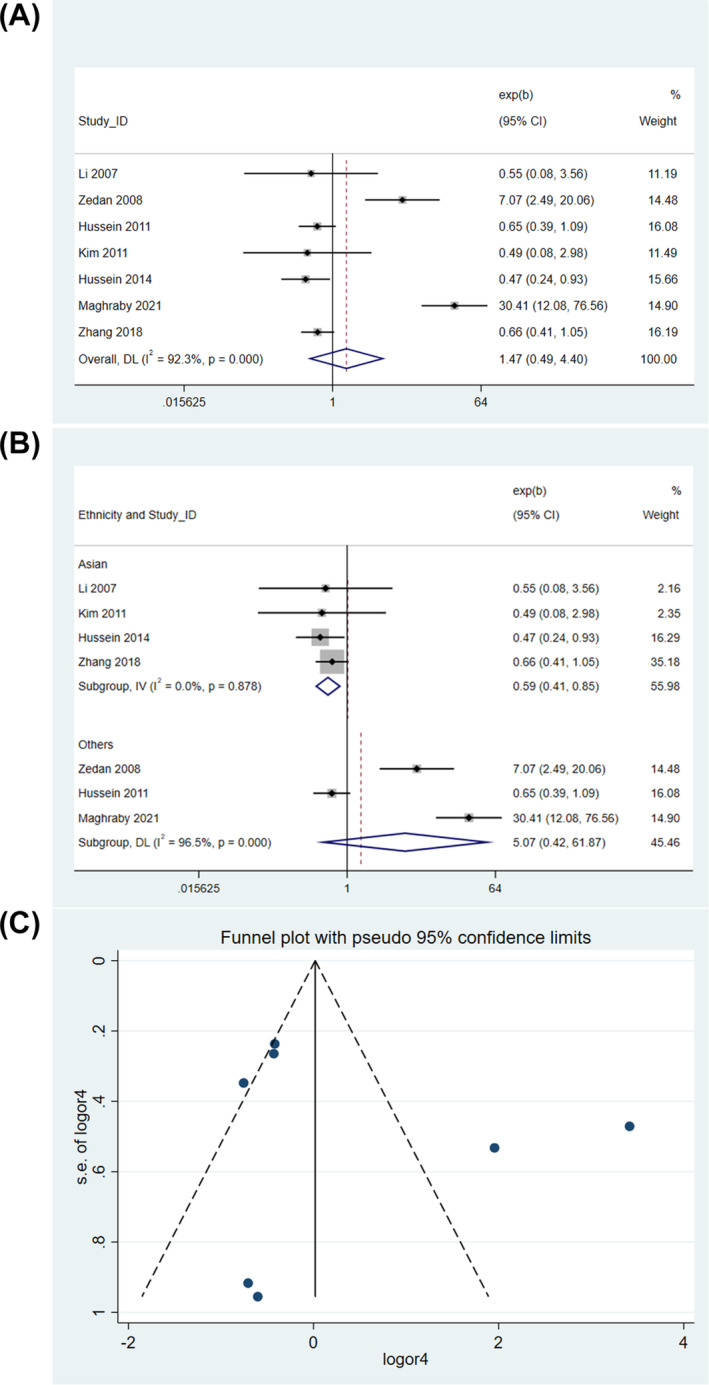
Genetic association in recessive model. (A) Overall association. (B) Subgroup analysis by ethnicity. (C) Funnel plot.

When the data were analyzed in the recessive model, no significant association was found between the polymorphisms and the risk of pediatric asthma (random‐effects model, OR = 1.475, 95% CI = 0.495–4.397, *P* = 0.486) (Figure 5A). Removal of Maghraby et al^17^ from the analysis reduced the OR to less than 1, but the result was still not statistically significant (Supporting Information). Subgroup analysis showed that the *IL10* rs1800896 polymorphism was significantly associated with a reduced risk of pediatric asthma in Asians (fixed‐effects model, OR = 0.585, 95% CI = 0.405–0.846, *P* = 0.004), but no significant association was observed in Egyptians (Figure 5B). As shown by Egger's test (*P* = 0.422) and inspection of the funnel plot, no publication bias was detected (Figure 5C).

### 3.6 Meta‐analysis findings: Allele model

**FIGURE 6:**
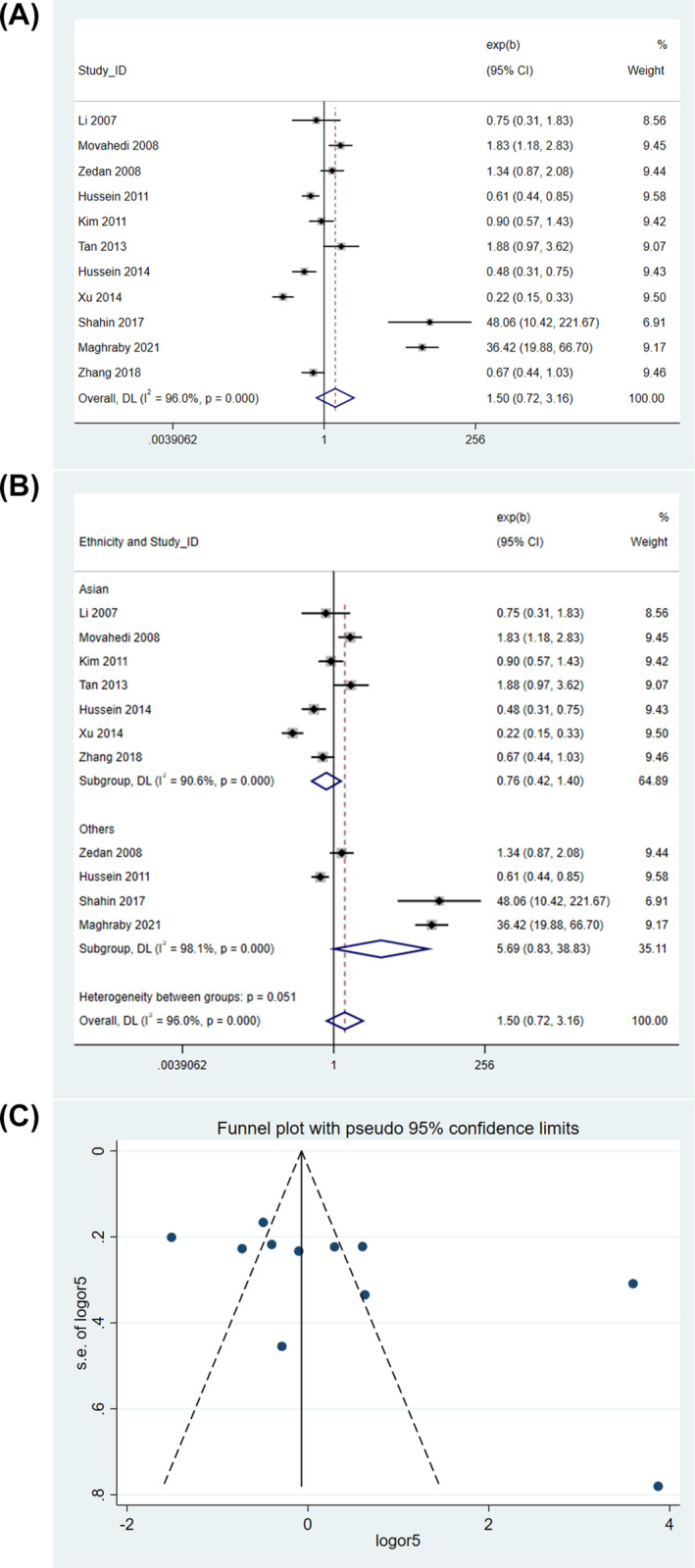
Genetic association in allele model. (A) Overall association. (B) Subgroup analysis by ethnicity. (C) Funnel plot.

In the allele model, pooled data from the included studies demonstrated a lack of significant relationship of the polymorphism with susceptibility to pediatric asthma (random‐effects model, OR = 1.503, 95% CI = 0.715–3.161, *P* = 0.282) (Figure 6A). The absence of significance was also observed in the Asian and Egyptian groups when subgroup analysis was performed according to ethnicity (Figure 6B). Sensitivity analysis showed that the results were not significantly altered by any of the studies (Supporting Information). No evidence of publication bias was detected based on the inspection of the funnel plot and the results of Egger's test (*P* = 0.082) (Figure 6C).

## 4 DISCUSSION

IL10 is widely recognized for its crucial involvement in the pathogenesis of asthma. However, studies exploring the association between the IL10 rs1800896 polymorphism and asthma in children have yielded conflicting outcomes, contributing to the inconsistency in the current understanding. The aim of this study was to resolve these contradictions through a meta‐analysis. Overall, no association was found between the polymorphism and asthma risk in children. However, when a subgroup analysis according to the ethnic background of the subjects (Asians vs. others; the latter subgroup consisted exclusively of Egyptians) was performed, it was found that polymorphism could significantly reduce the pediatric asthma risk in Asians in the homozygous and recessive models, and significant association was not observed for the Egyptian subgroup. The different observations between different subgroups are not surprising. Many genetic association studies have found significant associations in one ethnic group but not in the others.^24^, ^25^, ^26^, ^27^, ^28^, ^29^ This differential association underscores the interaction between genetic and nongenetic factors in the development of a disease.^30^ Indeed, longitudinal studies have shown that ethnic‐related differences are present in asthma risk and its outcomes.^31^, ^32^, ^33^ Ethnic‐specific analyses may therefore provide insights into the biological mechanisms of pediatric asthma in specific populations. It is known that the A allele of the *IL10* rs1800896 polymorphism results in lower mRNA levels of IL‐10 in vitro.^34^ Therefore, our results suggest that Asian children are better protected from pediatric asthma when IL‐10 levels are low. Further studies are needed to elucidate the mechanisms by which low IL‐10 levels protect Asian children from pediatric asthma, but this effect is not evident in Egyptians.

It is important to emphasize that our search strategy and eligibility criteria were unbiased with respect to geography. We used the major databases and searched specifically for terms related to IL‐10 and asthma. Our criteria, discussed in more detail in Section 2, were based on methodologic rigor and relevance to the study's content, without considering the origin of the research findings. Despite our unbiased approach, we found that all studies that met our eligibility criteria were from Asia and Egypt. This unexpected finding suggests that there may be an increased research interest in the association between *IL10* rs1800896 polymorphisms and pediatric asthma in these regions. The apparent geographic skew underscores the importance of further studies from different global contexts.

In the last decade, several meta‐analyses have been performed to examine the relationship between rs1800896 and asthma risk. The earliest work in this field was presented by Zheng et al,^35^ who showed a significant association in the recessive model after combining data from 2448 cases and 2146 controls. However, it should be noted that the work by Zheng et al included both adult and pediatric asthma cases, whose pathogenesis is known to be very different.^36^ Indeed, when Zheng et al performed a subgroup analysis by age (adults vs. children), no significant association was observed, which was consistent with our results. Our results were also in partial agreement with the previous meta‐analysis by Huang et al,^37^ which demonstrated no overall relationship between the rs1800896 and pediatric asthma risk, but a significant association in Asians under the dominant model and in non‐Asians under the recessive model, based on data from 1436 cases and 1112 controls. Another recent meta‐analysis by Dastgheib et al,^38^ which included 1298 cases and 1079 controls, also showed no statistical significance for the association between polymorphism and pediatric asthma risk. However, a subgroup analysis based on the ethnic group showed a significant association between polymorphism and pediatric asthma risk in Asians in the homozygous model.^38^

The different number of studies and the number of study participants included in the different meta‐analyses could explain the differences in the results of the different meta‐analyses. Compared with other previous studies, our study included the largest number of pediatric cases and controls, which gave us a higher power of the study, partly because we included a Chinese database in our literature search. For this reason, our meta‐analysis included a few studies that had not been included in other previous works, such as the studies by Zhang et al^22^ and Zhen et al^23^ In addition, in contrast to Zheng et al,^35^ we included only pediatric subjects because there are major differences in the pathogenesis of asthma between adults and children, as careful data extraction in a meta‐analysis is important to ensure that conclusions and interpretations are reliable.^39^ These measures may help provide a more precise evaluation of the relationship between the polymorphism and asthma susceptibility in children.

This meta‐analysis has some limitations. First, among the included studies, we found that Shahin et al^18^ and Maghraby et al^17^ appear to be outliers because their data showed a high risk (OR > 20) for pediatric asthma associated with the variant allele in many genetic models. However, considering the small sample size, this inflated result is not unexpected.^40^ Nonetheless, the inclusion of their data is unlikely to affect the results because studies with small sample sizes are weighted low in the meta‐analysis. Indeed, our sensitivity analysis did not show that omitting these studies would have significantly altered the results. Second, even though this meta‐analysis included the largest number of subjects, the power to show a significant relationship may still be insufficient. In addition, the included studies only included participants from Asian and Egyptian ethnic groups. Therefore, the association of rs1800896 and susceptibility to pediatric asthma in other populations is unclear. Finally, interactions between several genes or between genes and nongenetic factors are known to be involved the development of asthma.^41^, ^42^, ^43^ However, these aspects were not investigated in the present study because data on these interactions were unavailable in the included studies.

In conclusion, the present meta‐analysis shows that the *IL10* rs1800896 polymorphism was not significantly associated with the risk of pediatric asthma overall. However, the polymorphism was significantly associated with a lower risk of pediatric asthma in Asians in the homozygous model and in the recessive model. Therefore, there is potential for rs1800896 to be used as a predictive marker for pediatric asthma in the Asian population. Nevertheless, future studies should focus on addressing the gaps and limitations of this study.

## AUTHOR CONTRIBUTIONS

Zhihong Zhou performed the literature search, extracted the data, performed statistical analysis, and wrote the initial draft. Hui Zhang performed the literature search, extracted the data and performed statistical analysis. Yuanhong Yuan conceptualized the study and edited the draft manuscript.

## CONFLICT OF INTEREST STATEMENT

The authors declare no conflicts of interest.

## ETHICS STATEMENT

Not applicable.

## Supporting information



Supplementary information

## Data Availability

All data were presented in this manuscript and the supplementary file.
