# Supplementary material for: Association between IL10 rs1800896 polymorphism and risk of pediatric asthma: A meta‐analysis
Source: Clin Respir J. 2023 Nov 8;17(12):1276–85. doi: 10.1111/crj.13714 (PMC10730469; doi:10.1111/crj.13714)
Supplement: Supplementary file 1 — Supplementary information [file CRJ-17-1276-s001.docx]

**Supplementary information**

Newcastle Ottawa scale

| **Study ID** | **Selection** | | | | **Comparability** | **Outcome** | | |
| --- | --- | --- | --- | --- | --- | --- | --- | --- |
|  | **1** | **2** | **3** | **4** | **5** | **6** | **7** | **8** |
| Li 2007 ^19^ | ★ | ★ |  | ★ | ★ ★ | ★ | ★ | ★ |
| Movahedi 2008 ^12^ | ★ | ★ |  | ★ | ★ ★ | ★ | ★ | ★ |
| Zedan 2008 ^13^ | ★ | ★ | ★ | ★ | ★ ★ | ★ | ★ | ★ |
| Hussein 2011 ^14^ | ★ | ★ |  | ★ | ★ ★ | ★ | ★ | ★ |
| Kim 2011 ^15^ | ★ | ★ |  | ★ | ★ ★ | ★ | ★ | ★ |
| Tan 2013 ^20^ | ★ | ★ |  | ★ | ★ ★ | ★ | ★ | ★ |
| Hussein 2014 ^16^ | ★ | ★ |  | ★ | ★ ★ | ★ | ★ | ★ |
| Xu 2014 ^21^ | ★ | ★ |  | ★ | ★ ★ | ★ | ★ | ★ |
| Shahin 2017 ^18^ | ★ | ★ |  | ★ | ★ ★ | ★ | ★ | ★ |
| Zhang 2018 ^22^ | ★ | ★ | ★ | ★ | ★ ★ | ★ | ★ | ★ |
| Zhen 2018 ^23^ | ★ | ★ |  | ★ | ★ ★ | ★ | ★ | ★ |
| Maghraby 2021 ^17^ | ★ | ★ |  | ★ | ★ ★ | ★ | ★ | ★ |

Sensitivity analysis – homozygous model

| **Study omitted** | **OR** | **95% CI** |
| --- | --- | --- |
| Li 2007 | 1.240 | 0.180 - 8.553 |
| Movahedi 2008 | 1.111 | 0.197 - 6.263 |
| Zedan 2008 | 0.946 | 0.128 - 6.968 |
| Hussein 2011 | 1.313 | 0.147 - 11.727 |
| Kim 2011 | 1.259 | 0.181 - 8.757 |
| Tan 2013 | 1.111 | 0.197 - 6.263 |
| Hussein 2014 | 1.442 | 0.187 - 11.155 |
| Xu 2014 | 1.111 | 0.197 - 6.263 |
| Shahin 2017 | 1.111 | 0.197 - 6.263 |
| Maghraby 2021 | 0.505 | 0.251 - 1.017 |
| Zhang 2018 | 1.341 | 0.202 - 8.908 |

Sensitivity analysis – heterozygous model

| **Study omitted** | **OR** | **95% CI** |
| --- | --- | --- |
| Li 2007 | 1.086 | 0.424 - 2.782 |
| Movahedi 2008 | 1.063 | 0.445 - 2.541 |
| Zedan 2008 | 1.223 | 0.473 - 3.163 |
| Hussein 2011 | 1.200 | 0.433 - 3.330 |
| Kim 2011 | 1.095 | 0.391 - 3.063 |
| Tan 2013 | 0.988 | 0.387 - 2.524 |
| Hussein 2014 | 1.217 | 0.465 - 3.189 |
| Xu 2014 | 1.336 | 0.579 - 3.084 |
| Shahin 2017 | 0.829 | 0.351 - 1.962 |
| Maghraby 2021 | 0.710 | 0.340 - 1.483 |
| Zhang 2018 | 1.134 | 0.456 - 2.820 |

Sensitivity analysis – dominant model

| **Study omitted** | **OR** | **95% CI** |
| --- | --- | --- |
| Li 2007 | 1.325 | 0.414 - 4.238 |
| Movahedi 2008 | 1.257 | 0.430 - 3.671 |
| Zedan 2008 | 1.411 | 0.438 - 4.546 |
| Hussein 2011 | 1.437 | 0.411 - 5.022 |
| Kim 2011 | 1.316 | 0.366 - 4.731 |
| Tan 2013 | 1.198 | 0.367 - 3.917 |
| Hussein 2014 | 1.481 | 0.456 - 4.812 |
| Xu 2014 | 1.622 | 0.546 - 4.818 |
| Shahin 2017 | 0.883 | 0.306 - 2.551 |
| Maghraby 2021 | 0.763 | 0.347 - 1.676 |
| Zhang 2018 | 1.403 | 0.457 - 4.312 |

Sensitivity analysis – recessive model

| **Study omitted** | **OR** | **95% CI** |
| --- | --- | --- |
| Li 2007 | 1.672 | 0.513 - 5.452 |
| Movahedi 2008 | 1.475 | 0.495 - 4.397 |
| Zedan 2008 | 1.133 | 0.364 - 3.531 |
| Hussein 2011 | 1.700 | 0.413 - 7.001 |
| Kim 2011 | 1.701 | 0.520 - 5.559 |
| Tan 2013 | 1.475 | 0.495 - 4.397 |
| Hussein 2014 | 1.810 | 0.495 - 6.620 |
| Xu 2014 | 1.475 | 0.495 - 4.397 |
| Shahin 2017 | 1.475 | 0.495 - 4.397 |
| Maghraby 2021 | 0.860 | 0.436 - 1.697 |
| Zhang 2018 | 1.696 | 0.402 - 7.152 |

Sensitivity analysis – allele model

| **Study omitted** | **OR** | **95% CI** |
| --- | --- | --- |
| Li 2007 | 1.609 | 0.730 - 3.547 |
| Movahedi 2008 | 1.483 | 0.656 - 3.354 |
| Zedan 2008 | 1.535 | 0.671 - 3.512 |
| Hussein 2011 | 1.676 | 0.712 - 3.943 |
| Kim 2011 | 1.599 | 0.699 - 3.662 |
| Tan 2013 | 1.475 | 0.666 - 3.270 |
| Hussein 2014 | 1.703 | 0.754 - 3.850 |
| Xu 2014 | 1.824 | 0.870 - 3.826 |
| Shahin 2017 | 1.161 | 0.557 - 2.423 |
| Maghraby 2021 | 1.007 | 0.598 - 1.696 |
| Zhang 2018 | 1.651 | 0.719 - 3.789 |
